# Loss of the Endothelial Glucocorticoid Receptor Prevents the Therapeutic Protection Afforded by Dexamethasone after LPS

**DOI:** 10.1371/journal.pone.0108126

**Published:** 2014-10-09

**Authors:** Julie E. Goodwin, Yan Feng, Heino Velazquez, Han Zhou, William C. Sessa

**Affiliations:** 1 Department of Pediatrics, Yale University School of Medicine, New Haven, Connecticut, United States of America; 2 Department of Internal Medicine, Veterans Affairs Hospital, West Haven, Connecticut, United States of America; 3 Vascular Biology and Therapeutics Program, Yale University School of Medicine, New Haven, Connecticut, United States of America; 4 Department of Pharmacology, Yale University School of Medicine, New Haven, Connecticut, United States of America; University of São Paulo, Brazil

## Abstract

Glucocorticoids are normally regarded as anti-inflammatory therapy for a wide variety of conditions and have been used with some success in treating sepsis and sepsis-like syndromes. We previously demonstrated that mice lacking the glucocorticoid receptor in the endothelium (GR ^EC KO^ mice) are extremely sensitive to low-dose LPS and demonstrate prolonged activation and up regulation of NF-κB. In this study we pre-treated these GR ^EC KO^ mice with dexamethasone and assessed their response to an identical dose of LPS. Surprisingly, the GR ^EC KO^ mice fared even worse than when given LPS alone demonstrating increased mortality, increased levels of the inflammatory cytokines TNF-α and IL-6 and increased nitric oxide release after the dexamethasone pre-treatment. As expected, control animals pre-treated with dexamethasone showed improvement in all parameters assayed. Mechanistically we demonstrate that GR ^EC KO^ mice show increased iNOS production and NF-κB activation despite treatment with dexamethasone.

## Introduction

Exogenous glucocorticoids (GCs), such as dexamethasone, are anti-inflammatory compounds which are used to treat a variety of chronic inflammatory conditions as well as acute septic shock and sepsis-like syndromes. Dexamethasone (DEX) exerts its effects through the glucocorticoid receptor (GR), a nuclear hormone receptor that is ubiquitously expressed in most cells of the body and widely conserved across species [1]. Previous studies in animal models have demonstrated that prophylaxis with DEX prior to the onset of sepsis can decrease production of inflammatory cytokines, such as TNF-α, and reduce morbidity and mortality [2], [3]. In human trials, steroids have been used with considerably less success in this setting for many decades, as the mechanisms by which they confer their anti-inflammatory effects are still not clear [4], [5]. Recent studies have also suggested that rodent models may not be ideal systems in which to recapitulate human sepsis [6], [7] and thus the role of steroids in these systems is not clear.

Previous studies have shown clearly that GR has cell specific roles. For example, deletion of GR in the central nervous system results in mice with profoundly altered hypothalamic-pituitary-adrenal (HPA) axes and tenfold elevated circulating corticosterone levels as well as reduced anxiety-related behavior [8]. We recently showed that mice with tissue-specific deletion of GR in the endothelium were almost completely protected from steroid-induced hypertension [9], yet had increased mortality and hemodynamic instability in response to relatively low-dose LPS [10]. Given the dramatic phenotype we observed when these mice were treated with LPS, here we studied the role of endothelial GR in the setting of DEX pre-treatment before the administration of an identical dose of LPS. We hypothesized that the absence of endothelial GR would be a critical mediator of DEX effectiveness in this model of LPS-induced sepsis.

In this study we show that the presence of endothelial GR is required for DEX to rescue the animals from LPS-induce morbidity and mortality, and furthermore that in most parameters studied, DEX and LPS together result in worse outcomes than LPS alone in mice lacking the receptor in the endothelium. We further show that administration of DEX and LPS in the absence of endothelial GR results in increased levels of TNF-α, and iNOS and increased activation of NF-κB. Thus, though DEX is administered systemically, the presence of endothelial GR is required to mediate its protective effects in this setting. These data potentially have direct applicability to the approach to sepsis in human patients.

## Materials and Methods

### Mice

Male mice, age 8–12 weeks, with average weights of 20–25 grams, with tissue specific excision of the endothelial glucocorticoid receptor, designated GR ^EC KO^ mice and littermate controls, designated GR fl/fl were used for experimentation as described [9]. These mice are fully congenic, having been back-crossed for more than 20 generations. Mice used in these experiments were treatment-naïve and housed in standard mouse cages with a maximum of 5 animals/cage. They were maintained in a standard environment with 12-hour light/dark cycles and with free access to food and water throughout.

GR ^EC KO^ mice and littermate controls were pretreated with DEX via intraperitoneal (IP) injection and then injected IP 2 hours later with a single dose of LPS. DEX injections were performed between 8 and 9 AM and then LPS was administered between 10 and 11 AM. The two mouse genotypes were treated simultaneously. All experiments were performed according to a protocol approved by the Institutional Animal Care and Use Committee at the Yale University School of Medicine, and were consistent with the NIH Guidelines for the Care of Laboratory Animals. Pain and suffering were minimized as much as possible.

For this survival study, to assess the degree of impairment and/or pain which resulted from this injection the following parameters were used: loss of mobility, failure to groom, and presence of diarrhea. If an animal was found to have 1 or more of these symptoms for more than 8 hours it was euthanized by an overdose of ether and cervical dislocation. Half lives of LPS are estimated to be anywhere from 60–120 min [11], so 8 hours would represent a minimum of 4 half lives when <10% of the LPS would still be predicted to be present. If the mouse was symptomatic at this time, it was deemed unlikely to recover spontaneously. Mice were monitored 3 times/day for 3 days after injection. Mice were kept in their original cages throughout the experiment.

### Drug injections

LPS from E. Coli strain O55: B5 (VWR) was dissolved in PBS and prepared fresh prior to each experiment. A dose of 12.5 mg/kg was used according to several previously published studies [12], [13]. Dexamethasone phosphate (MP Biomedicals) was dissolved in PBS and prepared fresh prior to each experiment.

### Serum measurements

Corticosterone measurement was performed by ELISA (Assay Designs) according to the manufacturer's instructions. Nitric Oxide was measured by the Total Nitric Oxide Assay Kit (Assay Designs) according to the manufacturer's instructions. Mouse IL-6 and TNF-alpha levels were measured by ELISAs from Pierce Biotechnology.

### Apoptosis determination

Tissue sections were mounted on glass slides and TUNEL staining was performed using the ApopTag Peroxidase In Situ Apoptosis Detection Kit (Chemicon International). Five fields from each slide were analyzed and quantified by Image J software. On other slides CD68 (Serotec) staining was performed to identify macrophages and images were also analyzed by ImageJ software. The same gray scale threshold was used for all images and the area of each tissue occupied by macrophages was calculated for 3 sections per sample and averaged.

### Blood pressure measurement

Male mice, 10–12 weeks old, were maintained under 1.75% (vol/vol) isoflurane anesthesia. The carotid artery was catheterized and both a bladder catheter and peripheral IV line for intermittent saline injections were placed. After a 30 to 60 minute equilibration period dexamethasone 2 mg/kg was administered IV followed 2 hours later by LPS 5 mg/kg IV. Mice were monitored continuously by oscillometric blood pressure measurement for 4–6 hours after injection.

### Western blot

Tissues were snap frozen in liquid nitrogen, pulverized, and resuspended in lysis buffer (50 mM Tris-HCl pH 7.4, 0.1 mM EDTA, 0.1 mM EGTA, 1% NP-40, 0.1% sodium deoxycholate, 0.1% sodium dodecyl sulfate (SDS), 100 mM NaCl, 10 mM NaF, 1 mM sodium pyrophosphate, 1 mM sodium orthovanadate, 1 mM Pefabloc SC, and 2 mg/ml protease inhibitor cocktail (Roche Diagnostics)). Cells were lysed on ice with lysis buffer. Protein concentrations were determined with the DC Protein assay kit (Bio-Rad Laboratories). Lysates were analyzed by SDS-polyacrylamide gel electrophoresis (PAGE) and immunoblotting. Primary antibodies used include the following: iNOS (Cayman) and GAPDH (Affinity Bioreagents). Secondary antibodies were fluorescence-labeled antibodies (LI-COR Biotechnology). Bands were visualized with the Odyssey Infrared Licor system

### NF-κB activation

NF-κB Activation was measured by the TransAM NF-κB p65 kit (Active Motif) according to the manufacturer's instructions.

### Cell culture

Mouse lung endothelial cells (MLEC) were isolated as described [14]. Mouse-specific control or GR siRNA (Qiagen) was used at a concentration of 50 nM to effectively knock down GR. Cells were either (1) untreated, (2) treated with DEX alone (1 µM) for 24 hours, (3) treated with TNF-α alone (10 ng/ml) for 24 hours or (4) simultaneously treated with both DEX and TNF-α for 24 hours.

### Quantitative PCR

Total RNA was isolated from cells using the RNeasy mini kit (Qiagen) according to the manufacturer's instructions. RNA was revere transcribed using the Taqman Reverse Transcriptase kit (Applied Biosystems). qPCR was performed on a Bio-Rad iQ5 machine using the resultant cDNA, 2× SA Biosciences RT^2^ qPCR Master mix and gene specific primers. Primers used for the detection of mouse GRα were: 5′ AAAGAGCTAGGAAAAGCCATTGTC 3′ and 5′ TCAGCTAACATCTCTGGGAATTCA 3′ and for mouse GRβ were: 5′ AAAGAGCTAGGAAAAGCCATTGTC 3′ and 5′ CTGTCTTTGGGCTTTTGAGATAGG 3′. A common forward primer was used as described [15]. Gene expression was normalized to the housekeeping gene 18 s.

### Statistical analysis

Data are presented as mean ± SEM. Survival statistics were calculated by the Mantel-Cox test. Serum parameters, tissue macrophage expression levels, and apoptosis levels were evaluated by one-way ANOVA with Bonferroni's posttest. Blood pressure and hemodynamic data was evaluated by repeated measures ANOVA with Bonferroni's posttest. Statistical significance was set at a p value <0.05.

## Results

### GR ^EC KO^ mice are not rescued by dexamethasone after induction of sepsis by LPS

Endothelial GR deficient mice (GR ^EC KO^) were generated as previously described [10].

**Figure 1:**
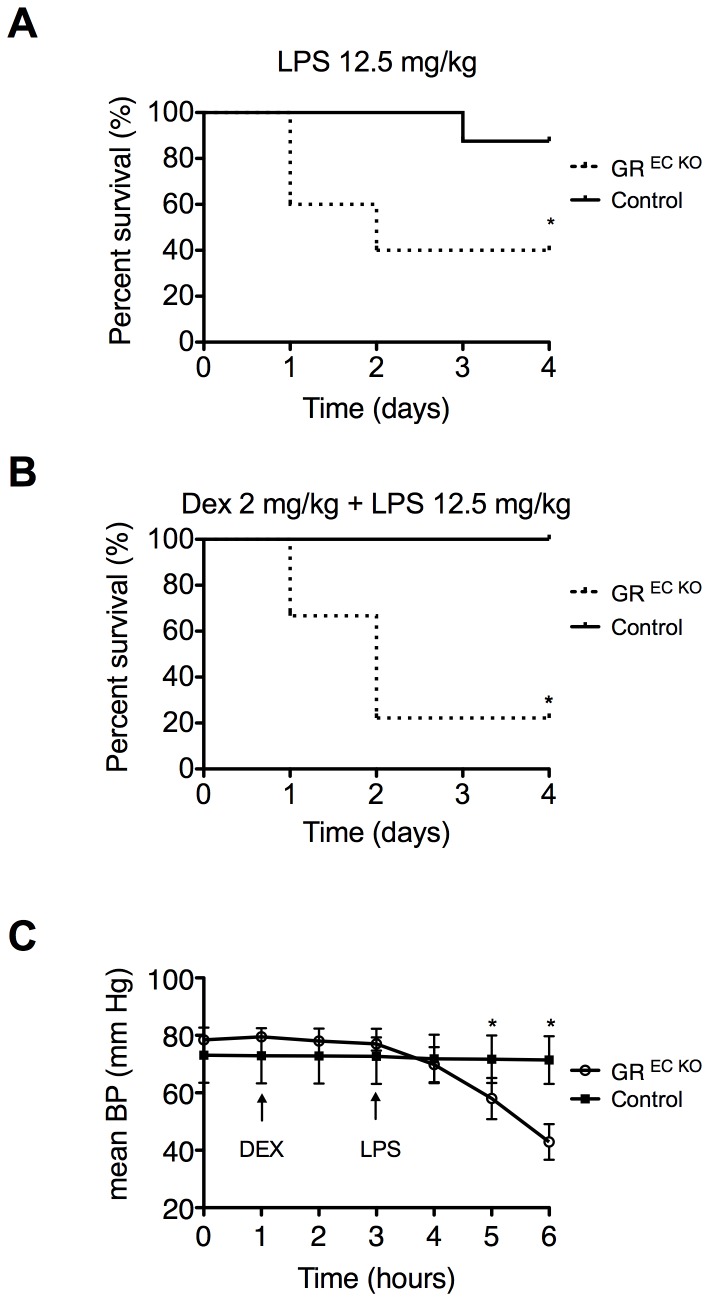
Impaired survival in GR ^EC KO^ mice after DEX. (A) GR ^EC KO^ mice show increased mortality after LPS treatment. (B) Mortality in GR ^EC KO^ mice is further increased in GR ^EC KO^ mice following DEX pre-treatment while controls are fully rescused following DEX+LPS. (C) Continuous blood pressure monitoring demonstrates hemodynamic instability in GR ^EC KO^ mice following pre-treatement with DEX while blood pressure is completely stabilized in control mice. *p<0.05

GR ^EC KO^ mice and Cre- GR fl/fl mice were pretreated with dexamethasone (DEX) (2 mg/kg IP) 2 hours before challenge with LPS (12.5 mg/kg IP) and survival was monitored over a period of 96 hours. We have previously shown that this dose of LPS resulted in greater than 50% mortality in GR ^EC KO^ mice, while produced very little mortality in controls [10]. We reproduced those experiments here again demonstrating that LPS-treated GR ^EC KO^ mice (n = 5) have 60% mortality while control animals (n = 8) show 12.5% mortality after an identical dose of LPS (Figure 1A). After pre-treatment with DEX, GR ^EC KO^ mice (n = 9) exhibited only 22% survival at the conclusion of the observation period compared to 100% of controls (n = 8) (p = 0.0031, Figure 1B). We have also previously demonstrated that this dose of DEX could rescue control animals treated with 80 mg/kg LPS [10]. GR ^EC KO^ mice demonstrated a more severe phenotype during the monitoring period which included decreased activity, decreased oral intake, shivering, diarrhea, and conjunctivitis.

### Dexamethasone does not stabilize BP in GR ^EC KO^ mice after acute LPS injection

To investigate the early hemodynamics in mice given DEX and LPS, continuous blood pressure monitoring was performed in anesthetized GR ^EC KO^ and control mice (n = 5/group) through indwelling carotid artery catheters. After a one-hour period of acclimation, DEX (2 mg/kg, IV) was administered and then LPS (5 mg/kg, IV) followed 2 hours later. As was previously observed in other experiments [9], there was a strong trend towards baseline mean arterial pressure (MAP) being slightly elevated in GR ^EC KO^ mice compared to controls (78.4±0.73 mm Hg vs. 73.1±1.91 mm Hg, p = 0.05). Three hours after LPS injection MAP in GR ^EC KO^ mice had fallen to 58.0±7.18 mm Hg while that of controls was relatively unchanged at 71.7±2.07 mm Hg (p = 0.03), and 4 hours after LPS injection MAP in GR ^EC KO^ mice was quite low at 42.9±6.17 mm Hg while controls were able to maintain their MAP at 71.4±2.07 mm Hg (p<0.01) (Figure 1C). These data show that loss of GR in the endothelium prevents hemodynamic stabilization by DEX.

### Dexamethasone is unable to suppress inflammation or nitric oxide release in GR ^EC KO^ mice

**Figure 2:**
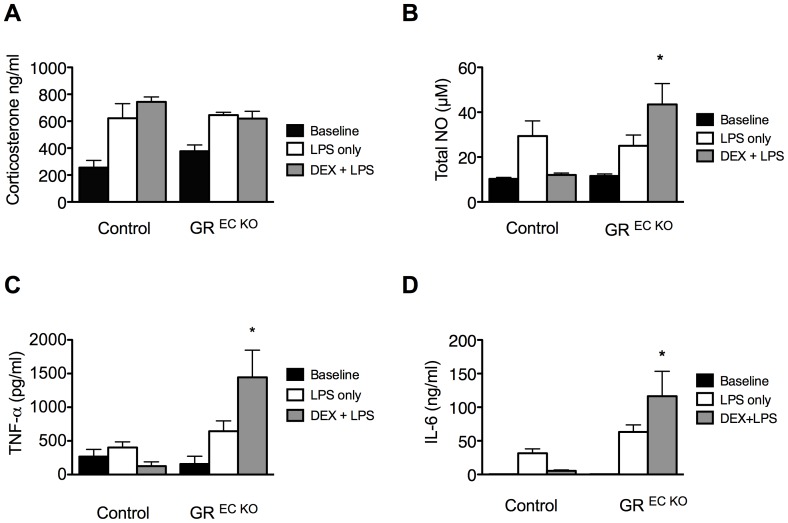
Heightened inflammation in GR ^EC KO^ mice following DEX pre-treatment. (A) No differences in corticosterone level were observed between GR ^EC KO^ mice and controls for any of the conditions tested. (B) Total nitric oxide levels in GR ^EC KO^ mice are increased following DEX+LPS. (C) TNF-α and (D) IL-6 levels are significantly increased in GR ^EC KO^ mice following DEX+LPS treatment while they are nearly unchanged from baseline in controls. All blood samples were collected 8 hours after LPS treatment (and 10 hours after DEX pre-treatment, if applicable). *p<0.05 compared to similarly treated controls.

In order to further investigate the increased mortality and hemodynamic collapse observed in the GR ^EC KO^ mice, corticosterone levels, nitric oxide levels and markers of inflammation were measured. All measurements were made in 4–6 mice/group. Endogenous corticosterone was measured at baseline and 8 hours after LPS treatment in mice that had been treated with LPS only and also those pre-treated with DEX and then given LPS. There was no difference in baseline levels; after LPS only, corticosterone levels were 622.1±108.2 ng/ml in control animals and 645.4±20.9 ng/ml in GR ^EC KO^ mice (p = NS). After DEX+LPS corticosterone levels were 743.6±37 ng/ml in controls and 619.2±54 ng/ml in GR ^EC KO^ mice (p = NS) demonstrating that the hypothalamic-pituitary-adrenal axis was functioning appropriately and release of corticosterone was similar in both groups under all conditions tested (Figure 2A).

Whole blood NOx (the sum of nitrite and nitrate) in plasma was measured in both groups either 8 hours after administration of LPS alone or 8 hours after LPS in those mice pre-treated for 2 hours with DEX. There were no differences in NOx levels at baseline. After LPS alone, control animals demonstrated a level of 29.4±6.7 µM while GR ^EC KO^ mice showed levels of 25±4.8 µM (p = NS). After DEX+ LPS, control mice demonstrated levels close to baseline at 12.0±0.9 µM, while GR ^EC KO^ mice showed markedly elevated levels at 43.5±9.2 µM (p = 0.015) (Figure 2B). It is important to note these levels exceed those in the GR ^EC KO^ mice treated with LPS alone.

We also measured levels of the inflammatory cytokines TNF-α and IL-6 at the same time point (Figure 2C, 2D) and observed a similar pattern. Both cytokines were able to be suppressed to near normal/undetectable levels in control animals pre-treated with DEX, while GR ^EC KO^ mice demonstrated levels that were significantly higher than those in mice treated with LPS alone, highlighting the fact that DEX is unable to suppress inflammation in GR ^EC KO^ mice challenged with LPS.

### Dexamethasone reduces apoptosis and macrophage recruitment in the liver, but not the lungs of GR ^EC KO^ mice

**Figure 3:**
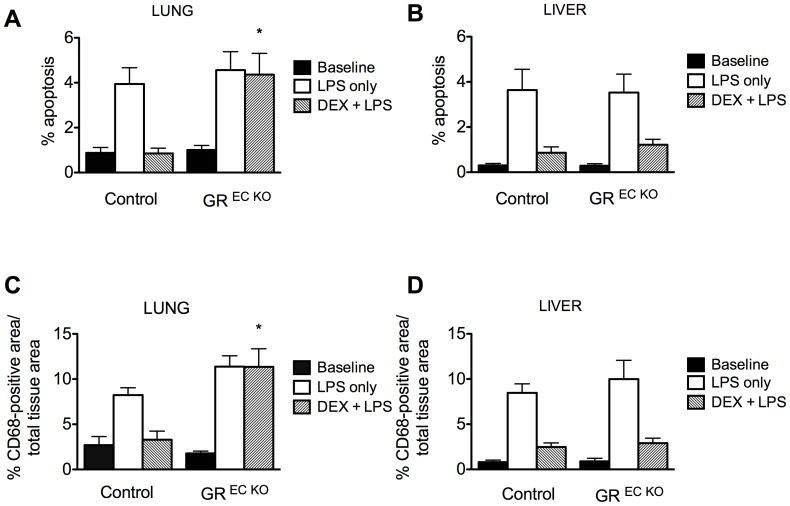
Lung apopotosis (A) and macrophage infiltration (C) are not improved by DEX in GR ^EC KO^ mice. Both GR ^EC KO^ mice and controls show an improvement in liver apoptosis (B) and macrophage infiltration (D) following DEX. *p<0.05 compared to similarly treated controls.

To examine the effects of DEX+LPS on specific tissues, apoptosis and macrophage recruitment were assessed by TUNEL staining and CD68 staining, respectively in the liver and lung of GR ^EC KO^ and control mice (n = 4/group). Organs were harvested 8 hours after LPS injection. In the lung, DEX was able to suppress apoptosis and lessen macrophage recruitment to near baseline levels in control animals but was ineffective in both regards in GR ^EC KO^ mice (Figure 3A, 3C). In contrast, DEX was effective in reducing apoptosis and macrophage recruitment in the livers of both GR ^EC KO^ and control mice (Figure 3B, 3D) implying that endothelial GR is not essential for hepatic protection by DEX.

### Dexamethasone increases iNOS expression and NF- κB activation in GR ^EC KO^ mice after LPS

**Figure 4:**
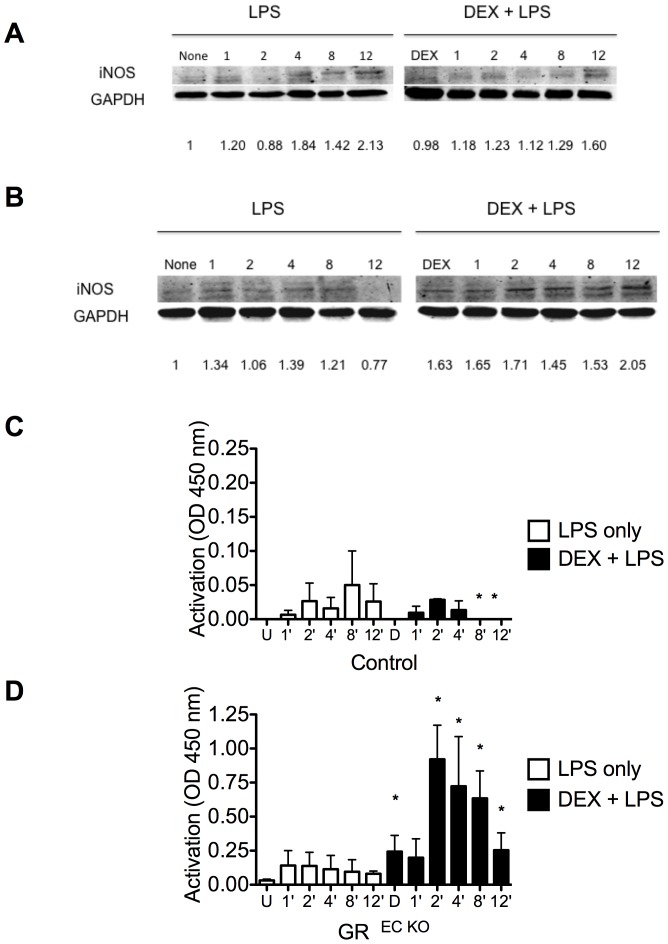
Increased iNOS expression and NF-κB activation in GR ^EC KO^ mice. Western blot of aortic homogenates from controls and GR ^EC KO^ mice treated with LPS alone or DEX+LPS and harvested at the indicated timepoints. Densitometry values are indicated below each lane. Activation of NF-κB was assayed in the same homogenates. (A) Control mice show decreased expression of iNOS when given DEX+LPS as compared to LPS alone and while (B) GR ^EC KO^ mice show increased iNOS levels following DEX+LPS as compared to LPS alone. (C) Activation of NF-κB is suppressed following DEX pre-treatement in control animals while in (D) GR ^EC KO^ mice increased activation of NF-κB is shown at every time point. *p<0.05 compared to similarly treated control. U = untreated, D = dexamethasone.

We previously showed that GR ^EC KO^ mice have increased iNOS expression after LPS [10]. To determine if pre-treatment with DEX affected the vascular control of iNOS, we pre-treated control and GR ^EC KO^ mice with DEX (2 mg/kg, IP) followed 2 hours later by LPS (12.5 mg/kg, IP) and harvested aortas over a range of time points to quantify iNOS protein levels. In control animals, LPS alone increased iNOS levels and pre-treatment with DEX reduced iNOS as shown by Western blotting at the time points examined (Figure 4A). In contrast, DEX increased iNOS protein levels in GR ^EC KO^ mice at all time points even up until 12 hours after LPS injection (Figure 4B). To determine if the increased iNOS expression was a result of enhanced NF-κB activation, we assayed the activity of the p65 subunit of NF-κB, which is accessible only when NF-κB is activated and bound to its target DNA. As shown in Figure 4C, pre-treatment with DEX was able to suppress NF-κB activation by 8 hours after LPS administration in the control animals, while in the GR ^EC KO^ mice DEX pre-treatment resulted in heightened activation of NF-κB at almost all time points tested (Figure 4D).

### Low expression of GRβ in endothelial cells

**Figure 5:**
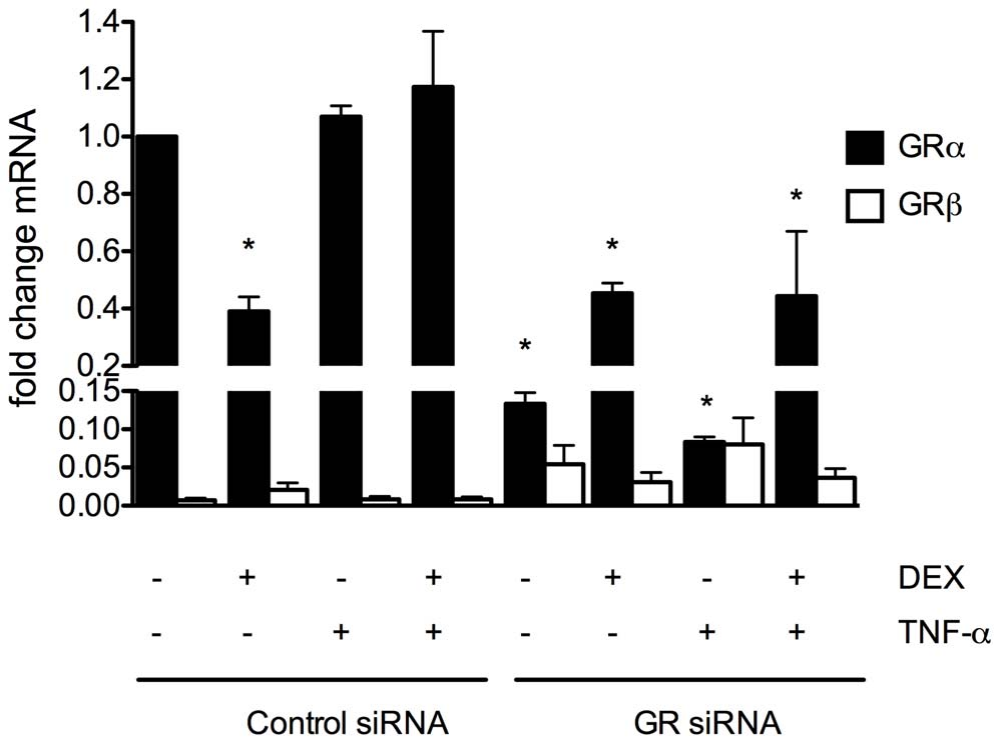
Expression profile of GRα and GRβ mRNA in endothelial cells. Real time PCR analysis was performed on mouse lung endothelial cells treated as described. Values were normalized to untreated control siRNA GRα levels and represent mean ± SEM for 3 independent samples. Cells were isolated from C57/BL6 mice. *p <0.05 compared to untreated control siRNA GRα levels.

The existence of mouse GRβ was only recently confirmed in 2010 and little is known about its role in physiology [15]. In humans there is evidence that GRβ can inhibit GRα resulting in glucocorticoid resistance. To assess whether up regulation of GRβ in endothelial cells could play a role in the poor response to DEX observed in our mutant mice, we measured GRα and GRβ mRNA by qPCR in mouse lung endothelial cells at baseline and after treatment with DEX only, TNF-α only, or a combination of both. As shown in Figure 5, GRα mRNA was significantly decreased in GR siRNA-treated endothelial cells as expected and was significantly decreased in DEX treated cells, also as expected. The GRβ isoform was present in very low abundance in all conditions tested (∼1–5%). There was a trend towards increased expression of GRβ after GR siRNA and DEX treatment though this did not reach statistical significance.

## Discussion

The major finding of this study is that selective elimination of endothelial GR prevents the therapeutic protection by DEX in the setting of LPS induced sepsis syndrome. Mechanistically this occurs by increased activation of NF-κB resulting in increased expression of iNOS, hemodynamic instability, and increased levels of inflammatory cytokines such as IL-6 and TNF-α. While our previous work has demonstrated that presence of endothelial GR is necessary for appropriate suppression of NF-κB [10], this study shows that absence of the receptor essentially mitigates and reverses the expected beneficial effects of DEX, which has been shown to prevent LPS-induced sepsis in several animal models [16]–[18]. Our study is particularly striking given that the expression of GR in all other cell types is unchanged, including in many immunologic cells germane to these studies such as macrophages, lymphocytes and neutrophils.

It is well known that glucocorticoids are effective therapy for many conditions mediated by NF-κB, a rapid response transcription factor involved in a host of immune and inflammatory conditions [19]. However, in our model the exogenous glucocorticoid not only is ineffective in rescuing GR ^EC KO^ mice following LPS but actually serves to worsen the phenotype. It should be re-iterated that GR ^EC KO^ mice demonstrate extreme sensitivity to LPS even in the absence of DEX, [10], a fact that highlights the importance of the endothelial GR since mice are traditionally viewed as an LPS-resistant species [20].

In trying to elcuidate how DEX promotes inflammation under our experimental conditions, it is important to consider the main mechanisms of how DEX is thought to suppress inflammation. Two hypothesis currently exist in this regard: (i) through direct protein interactions of GR with NF-κB and (ii) though interactions of GR with IκB, an inhibitory protein [19]. Our previous work has shown that elimination of endothelial GR results in prolonged activation of endothelial NF-κB in response to LPS [10]. Recent work has also suggested that the presence of the β isoform of the glucocorticoid receptor may be responsible for glucocorticoid resistance under some conditions, including in human septic shock [21], [22].

Hinds et al. discovered the existence of the β isoform of the glucocorticoid receptor in mice in 2010. As in humans, this isoform is unresponsive to the glucocortioid agonist DEX and is able to inhibit GRα under some conditions [15]. Though little characterization of this isoform has yet been performed in mouse, the relative abundance of the β isoform seems to vary with the tissue/cell type investigated, ranging from ∼30% of GRα in mouse embryonic fibroblasts and spleen to less than 5% in liver [15]. These results are in good agreement with human studies [23], [24] and the predominance in the spleen is consistent with the known dominant effect of GRβ in lymphoid tissue [25]. Haim et al. recently showed that LPS affects the levels of GR isoforms in bone marrow-derived macrophages, a cell type that is of great import in sepsis models [26]. These authors showed that incubation of these cells with 1 µM DEX resulted in a substantial decrease in the expression of both GRα and GRβ isoforms. Interestingly, in this study, treatment with LPS alone resulted in significant upregulation of both isoforms while treatment with DEX + LPS caused upregulation of GRβ and downregulation of GRα [26]. Endothelial cells have not previously been characterized though, based on our results, GRβ exists at very low levels in this cell type. Though there was a trend toward increased expression of GRβ with DEX and TNFα treatments in the GR siRNA-treated cells, it did not reach statistical significance. Of note, there was also a trend towards increased expression of GRα in the DEX-treated GR siRNA cells, which supports the ineffectiveness of DEX in suppressing inflammation observed *in vivo* in the knockout phenotype, though it is not clear if this may be cause or effect.

A number of recent publications have begun to report potentially deleterious effects of DEX in situations where they would normally be expected to be beneficial, raising the question of whether up regulation of GRβ under certain conditions may be playing a role. A recent study in the cancer literature notes that steroids unexpectedly promote cancer cell survival and induce chemotherapy-resistance in breast cancer [27], potentially analogous to the worsening septic phenotype we observe in the GR ^EC KO^ mice treated with DEX+LPS. These authors speculate that GR could interact with different NF-κB subunits which would result in regulation of genes through different NF-κB signaling pathways. A similar deleterious effect has been found in acute brain injury, whereby administration of steroids has been found to augment inflammation [28]. Interestingly one of the mouse models used in these studies was an endothelial GR knockout (using Tie-2 Cre) which clearly showed pro-inflammatory effects of steroids on the blood-brain barrier mediated through endothelial GR. However, in this study the expression of the GRβ isoform was not assessed. New data suggests that perhaps the anti-inflammatory actions of GR cannot be explained by a unifying mechanism but that characteristics of target genes and transcriptional state may provide situation-specific repression [29].

Finally it has been shown in *in vivo* models in both rats and mice that NF-κB activation can follow a biphasic response with early activation (6–24 hours) being associated with iNOS expression and pro-inflammatory cytokines and the late phase (>48 hours) being associated with absence of iNOS protein expression and paradoxical prolongation of inflammation [30]. Importantly, the NF-κB inhibitors used in this study were pharmacologic and not steroid-based. Though our experiments were performed during the ‘early phase’ and seem consistent with the pro-inflammatory response including iNOS activation, it is possible that the effects of DEX on NF-κB may include some dimerization that has not yet been characterized. Determining why DEX pre-treatment results in such a dramatic worsening of the phenotype in the GR ^EC KO^ mice will clearly require further study; however, it highlights that fact that mechanism of steroids' presumed ubiquitous anti-inflammatory action is clearly not well-understood despite their widespread clinical usage.

## Supporting Information

Checklist S1 **ARRIVE checklist.**
(PDF)
